# Characterization of pathogenic germline mutations in human Protein Kinases

**DOI:** 10.1186/1471-2105-12-S4-S1

**Published:** 2011-07-05

**Authors:** Jose MG Izarzugaza, Lisa EM Hopcroft, Anja Baresic, Christine A Orengo, Andrew CR Martin, Alfonso Valencia

**Affiliations:** 1 Structural Biology and Biocomputing Programme, Spanish National Cancer Research Centre (CNIO), C/Melchor Fernandez Almagro 3, E28029 Madrid, Spain; 2 Institute of Structural and Molecular Biology, Division of Biosciences, University College London, Gower Street, London WC1E 6BT, United Kingdom

## Abstract

**Background:**

Protein Kinases are a superfamily of proteins involved in crucial cellular processes such as cell cycle regulation and signal transduction. Accordingly, they play an important role in cancer biology. To contribute to the study of the relation between kinases and disease we compared pathogenic mutations to neutral mutations as an extension to our previous analysis of cancer somatic mutations. First, we analyzed native and mutant proteins in terms of amino acid composition. Secondly, mutations were characterized according to their potential structural effects and finally, we assessed the location of the different classes of polymorphisms with respect to kinase-relevant positions in terms of subfamily specificity, conservation, accessibility and functional sites.

**Results:**

Pathogenic Protein Kinase mutations perturb essential aspects of protein function, including disruption of substrate binding and/or effector recognition at family-specific positions. Interestingly these mutations in Protein Kinases display a tendency to avoid structurally relevant positions, what represents a significant difference with respect to the average distribution of pathogenic mutations in other protein families.

**Conclusions:**

Disease-associated mutations display sound differences with respect to neutral mutations: several amino acids are specific of each mutation type, different structural properties characterize each class and the distribution of pathogenic mutations within the consensus structure of the Protein Kinase domain is substantially different to that for non-pathogenic mutations. This preferential distribution confirms previous observations about the functional and structural distribution of the controversial cancer driver and passenger somatic mutations and their use as a proxy for the study of the involvement of somatic mutations in cancer development.

## Background

Point mutations of nucleotide bases are a mechanism of crucial importance in the evolution of proteins, and hence in the evolution of organisms. A biologically relevant class of point mutation, accounting for about 90% of sequence polymorphisms [1] at an overall frequency of about one per 1000 bases [2] is the single nucleotide point mutation or PM. Traditionally, polymorphisms are classified according to their genomic location into coding or non-coding. Coding PMs can be further classified depending on whether the resulting protein product is changed owing to the genomic polymorphism. Non-synonymous PMs (nsPMs) are those that alter the amino acid sequence of the protein product through either amino acid substitution or the insertion of truncation mutations. We refer to those which generate a single amino acid substitution as ‘single amino acid polymorphisms’ or SAAPs. In contrast, synonymous PMs (also referred as silent or sPM) are those that do not alter the amino acid sequence of the protein product expressed. A particular case of PMs corresponds to single nucleotide polymorphisms (SNPs): those germline mutations frequently found (>1%) in normal individuals and considered neutral. A major effort to catalogue and annotate SNPs is dbSNP [3]. Although most amino acid changes are tolerated in the native protein structure, not all PMs are neutral. An increasing number of mutations are prone to be associated with aberrant phenotypes and disease. Disease-associated mutations occur at much lower frequencies in the population and have a severe effect on phenotype. Here, we use the term ‘pathogenic deviation’ (PD hereafter) to refer to any single base change reported to be correlated with disease. Although both PDs and nsSNPs result in a change in the expressed protein product, the former are reported to have a severe effect on phenotype whereas nsSNPs are expected to have a non-deleterious phenotypic effect.

About 1% of all human genes are known to contribute to cancer as a result of acquired mutations. The family of genes most frequently contributing to cancer is the Protein Kinase gene family [4] which is implicated in a huge number of tumorigenic functions including immune evasion, proliferation, antiapoptotic activity, metastasis and angiogenesis, possibly due to the simplicity of the mechanism of attaching an ATP-derived phosphate to a substrate protein [5]. Protein Kinases are one of the most ubiquitous families of signaling molecules in the human cell, accounting for approximately 2% of the proteins encoded by the human genome [6]. Protein Kinases show a wide-scale similarity both at sequence and structure level, attributable to the fact that all kinases transfer the terminal phosphate of ATP to a serine, threonine or tyrosine residue in a target protein. Empirical studies to date also suggest a common, with a few exceptions, catalytic mechanism whereby ATP and an active site divalent cation are bound in identical manners and phospho-transfer is carried out by a shared set of amino acids [7]. Studies [8,9] on yeast models have shown that kinases can be very promiscuous, phosphorylating a huge number of different protein substrates albeit showing remarkable specificity. This inconsistency suggests that kinases have a region committed to the general function of catalysis, with another region (or regions) customizable to confirm substrate specificity to the enzyme without any particular need to alter fold, compromise ligand binding or modify the subsequent reaction mechanism. Protein Kinases are a thoroughly studied protein family and a plethora of mutations have been previously reported in the literature [10]. These studies often include evidence of association with disease. Concomitantly, several efforts [11,12] are devoted to the prediction of the pathogenicity of somatic kinase mutations in cancer samples. These mutations are classified into two main categories: those that are involved in cancer onset and development –driver mutations– and those that are biologically neutral –passenger– mutations. For a detailed review, see Baudot *et al.*,2009 [13]. Previous work [14] characterized the preferential distribution of cancer driver kinase somatic mutations in regions of importance for protein function, including disruption of substrate binding and/or effector recognition at family-specific positions, often avoiding structurally relevant positions.

The objectives of the work presented here are two-fold. Firstly, we wanted to clarify whether the trends detected for driver somatic kinase mutations can be extended to other disease related mutations independently of the nature of the mutation. Secondly, we wanted to provide additional information to the discussion on the interpretation of the role of kinase driver somatic mutations in the onset of cancer.

Consequently, we carried out a detailed multi-level comparative analysis of the differences between pathogenic and neutral (not pathogenic) germline mutations within the framework of the human kinome: amino acid composition of the polymorphisms was compared, mutations were characterized according to their potential structural effects and finally, we assessed the location of the different classes of polymorphisms with respect to kinase-relevant positions in terms of subfamily specificity, conservation, accessibility and functional sites.

## Results and discussion

### Sequence features of deleterious kinase mutations

We mapped 130 pathogenic deviations and 200 neutral SNPs to sequences within the Protein Kinase domain (PD*_PK_*s and SNP*_PK_*s, respectively). The native residue in the pathogenic (PD*_PK_*) set was enriched in glycines (p=0.01) and leucines (p=0.04) when the sequences were compared using a two-sided Fisher exact test whereas it was enriched in prolines (p=3x10^–5^) when the mutant amino acids were considered. As expected, in the SNP*_PK_* dataset none of the residue types were particularly enriched, neither in the native sequences nor in the mutated ones. Considering the native and mutant as a residue pair, three mutations were found more often in the PD*_PK_* dataset: leucine-proline (p=0), lysine-glutamate (p=0.02) and arginine-proline (p=0.02). Again, in the SNP*_PK_* dataset, no significant enrichment was found for the the wildtype-mutated pairs. The complete set of results can be found in Supplementary Table S2 in Additional file 1.

### Hypothesized structural effects of deleterious kinase mutations

SAAPdb [15] provides a characterization of the structural consequences of mutations. When these features were compared some differences between the groups appeared. PD*_PK_*s were often observed at the interface (p=0.03) including sites of inter-chain binding, as well as ligand binding. By contrast, SNP*_PK_*s significantly (p=0.04) more often than PD*_PK_*s tend to create empty spaces in the protein as a consequence of the difference in volume introduced by the side chain change, either in the core of the protein or in partially buried regions. Protein Kinase pathogenic mutations (PD*_PK_*) compared to pathogenic mutations outside this domain (PD*_nPK_*, standing for ’non protein kinase’) produced striking results: PD*_nPK_*s were more often explained by structural analyses: the modified residues affected stability, affected functional residues, introduced an empty region in the interior of the protein, and affected interaction prone positions (as annotated in MMDBBIND). By contrast, PD*_PK_* mutations were not significantly related with any of those categories. A complete description of these results can be found in Supplementary Table S3 in Additional file 1.

### Proximity of deleterious kinase mutations to kinase-specific protein features

**Figure 1:**
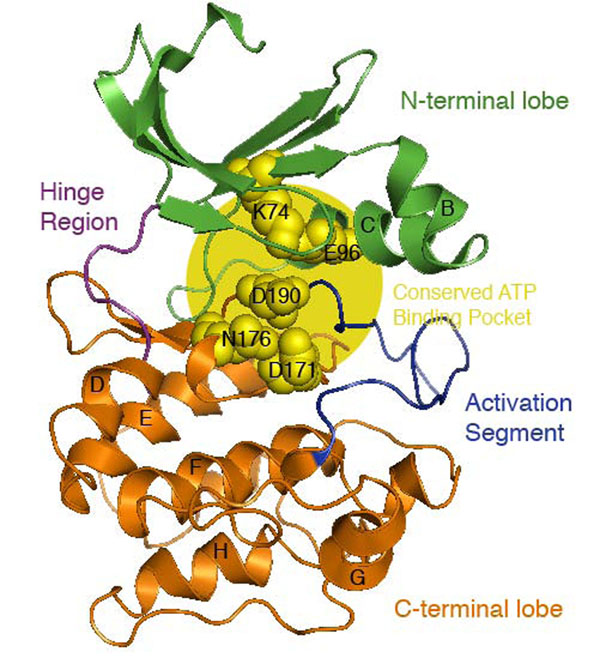
**Consensus model structure of summarizing the human Protein Kinase family** The model structure of human Protein Kinase, based on MAP3K1, shows the basic two-lobe kinase fold, with the N- and C-terminal (green and orange respectively) lobes joined by a hinge region (magenta). Substrate recognition is through interaction with the activation segment (blue), a region in the C-terminal lobe. The substrate-binding groove is located between the catalytic loop, the P+1 loop (activation segment), helix D, helix F, helix G and helix H. ATP binds at a site between the two lobes (yellow) that includes five conserved residues: (i) Lysine 74 that interacts with the alpha and beta phosphates of ATP and thereby stabilizing it; (ii) a nearby glutamic acid (E96) forms a salt bridge with lysine 74 increasing the stabilization network; (iii) Aspartate 171 is the catalytic base that initiates phosphotransfer by deprotonating the acceptor serine, threonine or tyrosine; (iv) Asparagine 176 interacts with a secondary divalent cation, thereby positioning the gamma-phosphate of ATP, and finally (v) Aspartate 190 which chelates the primary divalent cation, indirectly positioning ATP at the same time.

We present here the results of mapping the different types of mutations, PD*_PK_*s and SNP*_PK_*s, onto a representative structural model from the Protein Kinase superfamily. The mutations were analyzed in terms of their distribution relative to evolutionary conserved positions and known functional regions. We were able to map 47 positions containing at least one of the 62 PD*_PK_* mutations and 27 positions with at least one of the 36 SNP*_PK_*s to the consensus model (Figure 1) described in the Methods section and in previous studies [14]. The results of these analyses are summarized in Table 1.

**Table 1:** Results of the Xd analysis comparing PD*_PK_*s and SNP*_PK_*s

Feature	PD (Å)	SNP (Å)	∆Xd
Conservation - Shannon	7.17	6.71	-0.13
Conservation - AL2CO	8.49	10.49	-0.52
Structural Conservation	7.56	7.00	2.10
Accessibility - Buried	2.94	3.63	-0.83
Catalytic - FireDB	8.69	12.66	-4.74
Catalytic - Knight	11.89	16.26	-2.33
TreeDeterminants	6.00	7.94	-1.98

Where PD (Å) and SNP (Å) stand for the average closest distances in Angstroms from the feature residue and the PDs and SNPs respectively, and ∆Xd for the difference in Xd values. Negative values indicate that the PDs are closer to the feature residues whereas positive values indicate that SNPs are in the surroundings of feature residues.

#### Proximity of kinase mutations to known functional regions

##### a) Kinase mutations and the catalytic region

**Figure 2:**
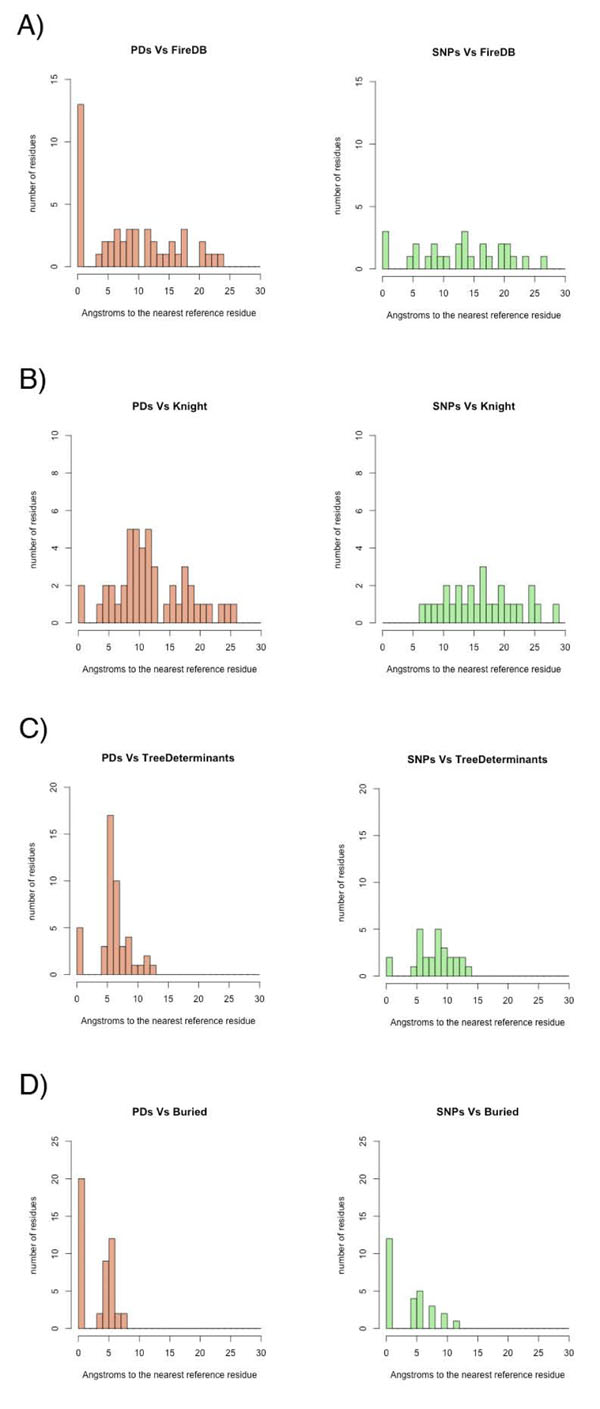
**Histograms of the distribution of distances between mutated resides and the analyzed features.** (A) Catalytic residues according to FireDB. (B) Catalytic Residues according to Knight et al (2007). (C) Tree-Determinants (D) Buried residues.

The active region of Protein Kinases includes the ATP binding site, the peptide-substrate binding sites and the catalytic loop implicated in the transference of the phosphate group. We defined the kinase-binding site as the set of residues extracted from the FireDB database [16]. This definition includes 32 residues (Figure 1) that directly contact the ATP in the binding pocket and that contains the five highly conserved residues that play a critical role in positioning ATP and stabilizing the active conformation in the catalytic mechanism [7] (see Figure 1). The distance distribution histograms (Figure 2, panels A and B) and the results in Table 1 (see also Figure S2 in Additional file 1, where the PD*_PK_*s, SNP*_PK_*s and catalytic residues were represented), showed a very strong tendency of PD*_PK_*s to locate if not in catalytic residues, at least close to them. Indeed, 13 out of the 32 residues in the binding pocket were annotated as pathogenic whereas only three were annotated as neutral. Moreover, 2 out of the 5 residues described as essential for the correct functioning of the ATP binding pocket [7] were annotated as PD*_PK_*s.

##### b) Kinase mutations and regions of functional subspecificity

In this study we used the position of the tree-determinant residues as a proxy for functionally important regions in Protein Kinases, particularly those related with the specific functions of each one of the subfamilies. Residues specific to the various subfamilies of Protein Kinases were identified for each of the eight subfamilies in which KinBase categorizes the human kinome [6]. Our recent implementation of the sequence-space approach, S3det [17] identified 32 unique positions in the model as containing information relevant for differentiating between subfamilies; that is, residues that tend to be conserved in the specific subfamilies and vary to different degrees in the others (Fig. S3 in Additional file 1 depicts the distribution of the mutations along with these tree-determinant residues). Out of the 32 tree-determinants in the model, five were annotated as pathogenic (residues 50, 173, 190, 217 and 233 in the generated structural model) whereas only two were annotated as neutral. Pathogenic deviations, if not exactly in positions that were disease-associated, clustered around tree-determinant residues in general. This tendency was especially relevant for tree-determinants in the ATP binding pocket, but also appreciable in the other function specific tree-determinants. PD*_PK_*s were closer to tree-determinant positions than neutral SNP*_PK_*s (Fig. 2C) This was also clear in the difference of Xd values (-1.98) indicating the existence of significant differences between PD*_PK_*s and SNP*_PK_*s with respect to proximity to positions characterized as important for the function and subspecificity of the kinases.

#### Proximity of kinase mutations to the protein core

With the accessibility parameters defined in Methods, 99 residues were classified as buried in the kinase structural model; 20 of these buried residues are annotated as PD*_PK_*s and 12 were annotated as SNP*_PK_*s (three residues – 58, 197 and 218 – are described in both datasets) (Fig. S4 in Additional file 1). The distribution of distances (Fig. 2D) manifests a clear tendency of PD*_PK_*s to be closer to buried residues. As a matter of fact, the analysis of the mean distances of mutated positions to buried residues (2.94Å and 3.63Å respectively) revealed a tendency for the pathogenic deviations (PD*_PK_*s) to be closer to buried residues than the neutral polymorphisms. This fact was supported by a Xd difference of -0.83.

### Examples of well-characterized disease-associated mutations affecting kinase function

The statistically significant results provided after analyzing the sequence features of the single amino acid polymorphisms (see sequence features of deleterious kinase mutations section) are supported by several examples in the literature. Karkkainen *et al.*, (2000) [18] associated human hereditary lymphedema, a particular case of lymphatic obstruction, with mutations in the vascular endothelial growth factor receptor 3 (VEGFR-3). The endothelial cells lining blood and lymphatic vessels depend on signal transduction mediated by specific receptor tyrosine kinases for their differentiation into a primary vascular plexus (vasculogenesis) and for the sprouting and splitting of new capillaries from previously existing vessels (angiogenesis). Two mutations were reported as the cause of the disease, due to the loss of tyrosine kinase activity and hence impaired downstream signaling, and a slower rate of internalization of the receptor. The suggested model highlights a mutation in the second arginine in the highly conserved HRDLAARN motif in the catalytic site to proline that facilitates the hydrogen bond between aspartate and a hydroxyl group from ATP at the binding pocket. This aspartate is critical for protein function as it is believed to act as the catalytic base in the phosphotransfer reaction. Moreover, their study revealed that a mutation from leucine to proline disturbed protein function due to both a disturbance in protein structural integrity and an interference of ATP binding. Since the pathogenic residue is located in the middle of a *β*-strand, introducing a restricted mobility residue such as proline disrupts the interaction with the surrounding *β*-strands, destabilizing the protein fold in the region. In addition, the sidechain of this leucine is part of the adenine-binding pocket, therefore alterations in such a relevant position modify the shape of the cleft and might interfere with ATP binding. With respect to the significant lysine-glutamate mutation, Mao *et al.*, (2001) [19] described the commonly cited mutation K430E (amongst others) in Bruton’s tyrosine kinase (BTK, Q06187), speculating with regard to how it might cause severe XLA (X-linked agammaglobulinemia), and providing a solved crystal structure. BTK is expressed on the surface of B cells and its kinase activity is crucial in proliferation and differentiation to mature B lymphocytes. According to their mechanism, upon the trans-phosphorylation of Y551, the highly conserved K430 facilitates a hydrogen bond with E445, causing a shift of the αC helix. The K430E mutation disables the αC helix repositioning, which is crucial for the catalytic activity of BTK, hence impairing the creation of mature B cells.

### Assessing the possible functional role of relevant kinase mutations by their sequence-structure characteristics

Our general analysis of the distribution of mutations in Protein Kinases not only provided an overview of their relation with function and structure, but also provided an insight into their specific biomedical implications. In the work presented here, we summarized all the knowledge accumulated for the Protein Kinase domain in a single framework structure (Fig. 1) under the assumption that regions important for the structure/function of the kinases are common to the whole family and hence they can be used as a reference for the interpretation of the mutations in any of the individual kinases. The accumulation of information clearly increases the significance of the results provided and makes the distribution of the polymorphisms more reliable and accurate. For instance, mutations in the insulin receptor gene in humans (INSR) have been reported as disease-associated in the literature. Defects in INSR are the cause of insulin-resistant diabetes mellitus with acanthosis nigricans type A (IRAN type A, MIM:610549), a syndrome characterized by the association of severe insulin resistance manifested by marked hyperinsulinemia and a failure to respond to exogenous insulin with the skin lesion acanthosis nigricans and overian hyperandrogenism in adolescent female patients. The relation of the disease with mutations in kinases is thoroughly reported in OMIM. Recent studies have further characterized the relationship between insulin resistance and disease (for example, [20]). Moreover, several studies have associated acanthosis nigricans with mutations in other kinases, such as the fibroblast growth factor receptors II and III [21-23]. Here, we identified A1161T (residue 173 in the model), a well-characterized mutation that introduces an alanine-threonine shift in the ATP binding pocket of INSR. This mutation has been defined in our analysis as a pathogenic deviation (PD*_PK_*). Concomitantly, it is a catalytic residue in FireDB and is important for family specificity. This perturbation of the ATP binding pocket might explain the unpaired phosphorylation and therefore the reduced enzymatic activity leading to the aberrant phenotype.

Finally, we considered not only a single mutation but a pair of consecutive mutations: T341P and C342F (positions 233 and 234 in the structural model respectively), in the human fibroblast growth factor receptor type 2 (FGFR2) to demonstrate that although only T341P is reported to be a pathogenic deviation in our dataset, the analysis can provide insights into complex diseases caused by more than one mutation at the same time. Several diseases caused by uncontrolled cell growth have been associated to defects on FGFR2. Among them, two related syndromes – Pfeiffer syndrome (PS) and Crouzon syndrome (CS) – have been reported to be associated to the pair of mutations of interest (T341P in PS and CS, C342F in CS). In our analysis, we described T341P as a mutation that introduces a change from threonine to proline in a buried sequence-conserved position. Other authors [15,24] have shown that mutations to proline are very often associated with disease. Additionally, we have characterized mutation C342F as a replacement of a buried residue. In addition, we identified both mutations as tree-determinant residues, thus considered related to binding specificity and indicative of the importance of these two positions for protein function.

### Conclusions

We have analyzed point mutations in the structure of Protein Kinases in order to characterize the structural and functional singularities of pathogenic and neutral mutations. Although the definition of the groups is by no means stable and the groups are constantly being redefined as new studies on the pathogenicity of mutations arise, this might be used as a proxy to deepen the knowledge on the underlying mechanisms of disease. The human kinome is particularly amenable for this type of study since much is known about the structure and function of this protein family and very relevant cancer-associated mutations have been published for these proteins [11,12,14].

To address this point, pathogenic deviations mapped to the kinase domain (PD*_PK_*) and single nucleotide polymorphisms mapped to the kinase domain (SNP*_PK_*) were compared on the basis of sequence, hypothesized structural effect and proximity to known kinase-specific features within the framework of a modeled consensus structure, representative of the whole superfamily. At the sequence level, several mutations were differentially observed in the PD*_PK_* and SNP*_PK_* datasets: the leucine-proline mutation emerged as an interesting feature of the PD*_PK_* dataset: it was identified both when analyzing the native and mutant residues separately, and when analyzing the mutation pairs, and it has been identified as being indicative of disease elsewhere, both specifically in a kinase disease dataset [24] and in wider, non-disease-specific datasets [15,25,26]. In addition, replacing an arginine with a proline was more often observed in the PD*_PK_* dataset. Again, this mutation has been described as being associated both with diseases predicted to be related to mutations in kinases [24] and across all other diseases [15,26]. Finally, replacing the positive charge of Lysine with the bulkier, negatively charged glutamic acid sidechain is identified in the PD*_PK_* dataset. Unlike the well-characterized previous mutations, this is a novel observation. These finding provide evidence about the existence of distinctive differences between the two types of mutations even at such a coarse grain level as the amino acid composition.

In the second part of the analysis we focused on the singularities of the mutations in structurally and functionally relevant regions. Thus, we characterized PD*_PK_* mutations in terms of their structural consequences by comparing them to SNP*_PK_* mutations. PD*_PK_* mutations were more likely to occur at the interface with ligands and other protein chains and SNP*_PK_* mutations were more likely to introduce a cavity in the protein core. This is coherent with the previous publications concluding that disease-associated mutations in kinases often affect the site of ATP binding [16,18,19]. In addition, PD*_PK_* mutations were compared to other PD mutations (PD*_nPK_*) to comment on the mechanisms by which mutations in kinases might lead to disease. In general, it is easier to explain PD*_nPK_*s in structural terms. Most noticeably, very few PD*_PK_* mutations resulted in a significant cavity, an empty space, or affected protein stability at all. An increasing body of literature attributes the pathogenic nature of PDs to their destabilizing effect on native protein structure [15,27-30]. The results here, specific to the mutations in the Protein Kinase domain, contradict this trend, indicating that the pathogenicity of PD*_PK_*s could not be simply attributed to a decreased stability of the resulting proteins. This might be explained by the fact that Protein Kinases are known to be a highly structurally conserved superfamily, while varying significantly with respect to sequence; as such, the structures must be tolerant to sequence variation. Hence, considerable flexibility must exist within the protein structure to be robust to sequence diversity. It is noticeable the relatively small number of structures available, we are aware that the results will benefit from a dedicated modelling pipeline, especially for very divergent kinases differing from the canonical PK domain. By contrast, a customized model for every single protein would not only increment the complexity of the comparative analysis of distances but would also add some uncertainties derived from the use of protein models.

In order to provide more detailed insight into the distinctive implication of pathogenic mutations in the disruption of protein function, we characterized the differences between PD*_PK_*s and SNP*_PK_*s by their association to kinase-specific functional and structural features. There were significant differences between the two types of mutations in terms of conservation, accessibility, distance to active/binding site residues and distance to family specific binding sites. It is interesting to compare these results with the ones previously obtained for driver/passenger mutations in Protein Kinases [14] from the works by [11,12] that constitute a subset of the many mutations stored in COSMIC [31]. Drivers are those mutations predicted to be involved in cancer onset whereas passenger mutations are those that are supposed to be accumulated during cancer progression being neutral respect to the origin of the cancer. In the previous work, the main conclusion was that driver mutations tended to be located near to important residues such as sequence conserved positions, family specific regions and active/catalytic sites whereas passenger mutations were located closer to structurally conserved regions. The difference observed here for the set of PD*_PK_*s and SNP*_PK_*s mimicked the one previously observed for driver and passenger mutations. Both datasets proved to be non-redundant. Only 4 residues were common to both driver and pathogenic datasets, all of them in the human B-raf proto-oncogene. The small overlap encountered is consistent with the very different nature –germline and somatic– of the mutations in each of the disease-prone datasets. Thus, the new results presented here can be seen as a confirmation of the functional/structural role of the mutations that are more likely to be pathogenic (drivers and PDs). Given that the definition of mutations as drivers and passengers is somehow controversial (For a review, see [13]), it is good to see that the current results could be interpreted as an indirect support to the categorization of mutations into drivers (disease-associated) and passengers (disease-neutral) and their use as a proxy for the study of the involvement of somatic mutations in cancer biology.

In summary, we have confirmed that the pathogenicity of mutations within the Protein Kinase superfamily is related to essential aspects of protein function, including perturbation of substrate binding and recognition by effectors at family-specific positions. As a matter of fact, pathogenic deviations (PD*_PK_*) accumulate in key functional regions whereas they seem to be absent from structurally relevant positions. These observations reinforce the idea that the pathogenicity of disease-associated mutations can be attributed to a disruption of native protein function while avoiding drastic changes prone to disrupt the protein globally. This tendency was not observed for neutral polymorphisms, which are apparently less disruptive of protein function and tend to be tolerated. Consequently, they are more often found in normal individuals. However, it is clear that further classification of the mutations in more specific subgroups will be necessary to provide a deeper knowledge on the mechanisms leading to disease. A typical example would be the characterization of mutations into a gain-of-function/loss-of-function on a large-scale.

The analysis presented here provides not only a characterization of the mutations, but also in some cases additional insight into the specific biomedical implications of the mutations. This type of approach will be particularly useful as part of the bioinformatics platform developed for the International Cancer Genome Consortium and other cancer genome projects. The results discussed in this work are biologically sound not only because they contribute to our understanding of the role of mutations in disease, but also because they can bee seen as a necessary step towards the development of predictors of pathogenicity based on the combination of the features analyzed here. Indeed, further development of the idea presented here might naturally derive in a classifier that making use of machine learning techniques would be able to predict the pathogenicity of any novel kinase mutation.

Moreover, although out of the scope of this work, the methodology used to analyze the distribution of the mutations in relation to a set of features can be applied to other families. However, we see (as other authors in the field [24]) advantages to the in-depth exploration of a family with more information about sequence, structure and mutations and for what it is feasible to obtain phylogenetic information to calculate subfamily specific sequence features. It remains interesting to analyze other protein families at a genome wide scale to corroborate to what extend the results shown in this work can be generalized.

## Methods

### Protein Kinase domain sequences

The KinBase resource [6] is a repository of the currently accepted classification of eukaryotic Protein Kinases. At the moment of the analysis, KinBase contained 620 human protein sequences of which 516 were Protein Kinases not defined as pseudogenes by the database curators. Although it has been described that some kinase pseudogenes are transcribed and even might have a residual or scaffolding function [32] kinase pseudogenes were not considered in the analysis performed here. KinBase does not map directly onto UniProtKB [33]. The mapping was performed using a BlastP [34] for each sequence against a custom database containing all entries in UniProtKB annotated as Protein Kinase domain for human. We were able to map 488 KinBase identifiers to a valid UniProtKB entry, 474 of them (97.13%) at sequence identity levels of at least 95%.

### Classification of the mutations

SAAPdb [15] is a database of single amino acid polymorphisms (SAAPs) mapped to protein structure. SAAPdb aims to provide likely structural effects of mutations and identify differences in potential structural consequences between neutral and pathogenic mutations. The disease dataset is derived mostly from OMIM [35] whereas the neutral dataset comes from dbSNP. For the Protein Kinase domain of the 488 kinases in SAAPdb contains 130 pathogenic deviations (PD*_PK_*s) and 200 neutral polymorphisms (SNP*_PK_*s). Of these 130 PD*_PK_*s, 62 were successfully mapped to a residue in the solved PDB structure. Similarly, of the 200 SNP*_PK_*s mapped to sequence, 36 were mapped to a PDB structure (See Supplementary Table S1 in Additional file 1). In order to create control datasets 9263 non-kinase PDs (PD*_nPK_*) were retrieved from SAAPdb, out of them 4652 mapped to a structure. All three datasets contain only unique, non-synonymous (missense) mutations. We excluded nonsense and synonymous mutations because these have a known, truncating effect or no effect on the protein structure. A unique mutation was defined by the combination of four parameters: UniProtKB accession number and sequence position, native amino acid and mutated amino acid.

### Comparing mutations with respect to the native and/or mutant residues

To compare the mutations with respect to their sequence features, the native and mutant residues were extracted from SAAPdb, as described above. Two-sided Fisher exact tests were carried out since they allow robust comparison of datasets of disparate sizes and evaluation of contingency tables with empty cells.

### Structural effects of mutations

The SAAPdb hypothesized structural effects are fully described elsewhere [15] and are summarised briefly below. (a) Mutations affecting stability: mutations on the surface of proteins that replace a hydrophilic residue with a hydrophobic residue are identified as introducing *unfavourable hydrophobicity on the surface*. Similarly, mutations in the core that replace a hydrophobic residue with a hydrophilic residue are identified as *introducing unfavourable hydrophobicity in the core*. Buried mutations that create a *charge shift* are also identified. Using a geometric analysis of PDB structures, SAAPdb identifies mutations that affect potential *disulphide bonds*. Mutations that introduce large *cavities* in the protein core or that break *hydrogen bonds* are identified as well. (b) Mutations affecting folding: unfavourable (with respect to torsion angles) mutations from *cis-proline*, *from glycine* and *to proline* are identified. Mutations that will *clash with existing residues* are identified. (c) Mutations to UniprotKB annotated residues: mutations at the site of residues annotated by UniprotKB as functionally relevant. (d) Mutations to binding residues: PDB structures are analysed to identify residues *binding* to proteins, DNA or small molecules. These data are augmented by data from *MMDBBIND*. (e) Mutations disrupting the quaternary structure (f) Mutations to sequence conserved residues. In addition, we provide results for the summary analyses categories of *structurally explained* (where at least one structural explanation is true, i.e., any explanation apart from the sequence conservation) and *explained* (where at least one explanation is true). In order to compare the mutations with respect to their structural effects, a binary explanatory vector is calculated for each mutation and a two-sided Fisher exact test was carried out.

### Generation of a consensus model summarizing Protein Kinase structures

A consensus model (Fig.1) of the basic structure of the kinase domain was created. This consensus model represents the average structure of a large number of kinases widespread along the human kinome, and therefore it is useful to summarize global characteristics of the structures. To build the model we first selected MAP3K1 as a standard representative sequence of the family from a manually curated multiple sequence alignment of the human kinome constructed using the alignment package MUSCLE [36]. The selected sequence was submitted to Modeller [37] assembling the models created using all those closely related template PDBs structures returned from a BLAST search. The predicted model has previously been used as a consensus of the Protein Kinase domain [14]. Finally, mutations were transferred from their own PDB coordinates to the consensus model for comparison.

### Calculation of important regions

#### Calculation of accessibility

NACCESS (Hubbard, *unpublished*) is a stand-alone program that calculates accessible areas by rolling a probe with van der Waals radius over the surface of the molecule. A residue is defined as buried if 16% or less of the residue’s surface is exposed to the probe. This is a common threshold [38] that ensures a reasonable number of buried residues.

#### Definition of catalytic sites

The FireDB database [16] contains a comprehensive curated set of substrate binding and catalytic residues, extracted directly from the PDB [39] or from the Catalytic Site Atlas [40]. FireDB binding residues for the various kinases were mapped into the general model using the corresponding multiple structure alignments.

#### Prediction of Tree Determinant positions

S3det [17] is an algorithm for the detection of groups of proteins within a family with potential functional specificities and to identify the residues that are characteristic of that group. S3det is based on the simultaneous quantitative analysis of sequences and residues within a multiple sequence alignment on related multidimensional spaces. Those residues associated with specific sequence subfamilies tend to be determinants of functional specificity and are located in functional regions of protein families, including substrate binding sites, functional sites and protein interaction sites.

### Xd analysis

To assess the significance of the proximity of different sets of mutations to areas of the protein (buried, functional, conserved, etc) we used the harmonic deviation, Xd, measure introduced previously [41].(1)

Where n is the number of distance bins in the distributions, d*_i_* is the upper limit for each bin, P*_ic_* is the percentage of residues with distance between d*_i_* and d*_i-1_* and P*_ia_* is the same percentage for all residues in the protein. Defined this way, positive values of Xd indicate that the population of residues is shifted to smaller distances with respect to the population of all residues. In practice we used a difference of Xd values of 0.75 to indicate distributions of residues that are significantly different regarding their proximity to previously defined areas of the protein. This threshold – albeit arbitrary – is based on manual inspection of previous results and has been proved valid in a similar context [14].

## Competing interests

The authors declare that they have no competing interests.

## Authors contributions

AV, CO and AM conceived the idea. All authors planned the analysis. JMGI, LEMH and AB generated the datasets. JMGI and LEMH performed the analysis. All authors discussed the results. JMGI, LEMH and AV wrote the manuscript. All authors read and approved the manuscript.

## Supplementary Material

Additional File 1 Click here for file
