# Supplementary material for: Characterization of pathogenic germline mutations in human Protein Kinases
Source: BMC Bioinformatics. 2011 Jul 5;12(Suppl 4):S1. doi: 10.1186/1471-2105-12-S4-S1 (PMC3194193; doi:10.1186/1471-2105-12-S4-S1)

Supplementary Materials

Proximity of kinase mutations to regions of sequence conservation.

The analysis of the conservation evaluated in terms of variability using AL2CO (Pei and Grishin, 2001) reveals 14 conserved residues, three of which are pathogenic deviations (positions 164, 216 and 233) and two of which are neutral SNPs (positions 49 and 228) (Figure S5). Visual inspection reveals that to some extent conserved positions tend to be surrounded by pathogenic deviations even if there is not an exact hit. A similar trend is observed when analyzing the results in terms of Shannon's Entropy, where one additional pathogenic deviation is identified as affecting a highly conserved residue in addition to the previous three (at position 54 in addition to 164, 216 and 233), and the same two neutral SNPs are identified as highly conserved (data not shown).

Histograms showing the distribution of the distances between mutated and conserved positions for the pathogenic deviations and neutral polymorphisms are represented in Figures S1(a) and S1(b), where PDPK mutations are in general closer to conserved residues than SNPPK mutations.

By contrast, Xd values depicted in Table 1 are not different enough to support this trend (XdSNPPK-XdPDPK(AL2CO)=-0.52 and XdSNPPK-XdPDPK(AL2CO)=-0.13 respectively).

Calculation of Conservation using Shannon's Entropy

For each position in the alignment conservation was measured in terms of Shannon's entropy (Shannon, 1948) which is a measure of the variability of the distribution of elements in a set as described by the formula:

$$-\sum_{i=1}^{n} p(x_i)\log_2 p(x_i) \quad (1)$$

where p(xi) is the probability of having element xi in bin i for that distribution. Conservation for each position in the alignment was measured by using 21 bins (on for each amino acid and one for gaps). As such, conservation here is equivalent to identity. Positions in the alignment were labelled as conserved if their Shannon's entropy was less than 0.20, and if the corresponding multiple sequence alignment contained less than or equal to 75% gaps.

Shannon CE. (1948) A mathematical theory of communication. The Bell Sytem Technical J.

Calculation of Sequence Conservation with AL2CO

AL2CO (Pei and Grishin, 2001) is a program to calculate a conservation index at each position in a multiple sequence alignment using several methods. Amino acid frequencies at each position are estimated and the conservation index is calculated for these frequencies. We used the AL2CO option to weight sequences to correct for unequal distances between the different sequence pairs in the alignment, and the matrix score that gives more weight positions occupied by residues with similar physico-chemical properties. Finally, we labelled as conserved those residues with a normalized conservation index threshold of 70%.

Pei J and Grishin NV. (2001) AL2CO: calculation of positional conservation in a protein sequence alignment. Bioinformatics 17 (8) 700-12

Proximity of kinase mutations to regions of structural conservation

Each fully-aligned position in the SSG alignment (see Methods) was scored for structural conservation using a modification of the method presented in [Orengo, 1999]. Those positions with a normalized conservation score better or equal to 8 were considered as conserved, resulting in 19 structurally conserved positions of which three were annotated as PDPKs and five as SNPPKs. Figure S6 depicts the distribution of the mutations along with the structurally conserved positions. Visual inspection of the image suggests that although not properly clustered around structurally conserved positions, SNPPKs tend to be slightly closer to conserved positions than PDPKs. This observation is supported by the histograms showing the distribution of distances (Figure S1(g)) and the differences in Xd values, which is of 2.10. (Table 1).

Calculation of Structural Conservation

The majority of kinases mapped to CATH superfamilies 1.10.510.10 and 3.30.200.20 (Phosphotransferase domain and Phosphorylase kinase respectively). In order to calculate structural conservation for each amino acid, it was necessary to generate a multiple structure alignment using CORA (Orengo, 1999). All 183 human kinase structures in the CATH superfamily were first clustered (complete-linkage) at 35% sequence identity to ensure that a sufficiently diverse set of kinases was used for the calculation, and representatives (S35Reps) taken from these 46 clusters. Although CORA is generally able to calculate accurate multiple structure alignments of all relatives from an entire superfamily, problems can occur in especially diverse folds where there is substantial structural variation. To assess the extent of structural diversity in the kinase superfamily, all S35Reps were aligned on a pair-wise basis using the SSAP structure comparison algorithm. The S35Reps were then grouped together if they shared Simax score < 3Å, producing four structurally similar groups (SSG). CORA was then used to multiply align all SSGs. Each fully-aligned position in the SSG alignment was scored for structural conservation using a modification of the method presented in Orengo, 1999. Vectors between C-beta atoms were calculated between a given pair of aligned residues in a given pair of structures, to all other equivalent positions (including gaps, which scored 0). The score for each alignment position was then calculated as a sum of the all the pair-wise protein scores. The score was then normalised across the whole alignment in the range 0-10, with 10 representing the most highly conserved positions. Positions with a conservation score of at least 8 were considered structurally conserved.

Orengo (1999). CORA--topological fingerprints for protein structural families. Protein Sci 8 (4) 699-715

Another example of well-characterized disease-associated mutations affecting kinase function
Another interesting example is the mutation D594G in B-raf proto-oncogene serine/threonine protein kinase (BRAF1_HUMAN). The RAF gene family consists of three members (ARAF1, BRAF, RAF1) each encoding serine/threonine kinases that are regulated by binding to RAS as part of the RAS-RAF-MEK-ERK-MAP kinase pathway which plays a critical role in cell proliferation and is frequently activated in cancer cells. Previous studies showed that mutated BRAF proteins have elevated kinase activity (Davies et al., 2002) and that the BRAF gene is somatically mutated in Non-Hodgkin lymphoma, a broad group of cancers affecting the immune system, indicating that the RAS-RAF kinase pathway in some NHLs may be regulated by somatic mutations of BRAF. In this study, we identified D594G as a pathogenic mutation (OMIM:164757.0011) introducing a Asp/Gly change (involved in the disruption of the binding site, interacting surface, quaternary structure and the essential scaffolding of hydrogen bonds) in the activation loop of the kinase family (position 190 in our model. Moreover, our analysis highlighted the importance of this amino acid as part of the ATP binding as described by FireDB and as a residue providing family specificity, this is, a tree-determinant. These descriptions are absolutely consistent with the important role of this mutation in the development of NHL as described above.

Davies et al. (2002) Mutations of the BRAF gene in human cancer. Nature 417 949-54

Supplementary Tables
Table S1: Summary of datasets used in the analysis
A summary of the three datasets used in the sequence analysis and the explanation vector analysis.

Dataset name	# of proteins	# sequence mapped	# structurally mapped
kdPD	31	130	62
kdSNP	125	200	36
nonkPD	1255	9263	4652

kdPD: the kinase domain PDs (non redundant, nonsynonymous)
kdSNP: the kinase domain SNPs (non redundant, nonsynonymous)
nonkdPD: the non-kinase PDs (non redundant, nonsynonymous)
proteins: the number of unique proteins (as defined by UniprotKB accession number) in the dataset
sequence mapped: the number of mutations mapped to protein sequence
structurally mapped: the number of mutations mapped to protein structure

Table S2(a): Comparing kdPDs and kdSNPs with respect to native residue

The results of the Fisher exact tests comparing kdPDs and kdSNPs with respect to the native residue in the mutation/polymorphism pair. Results are ordered by ascending p-value.

	kdPD		kdSNP		statistics		
**Native**	**n**	**%**	**n**	**%**	**p**	**set**	**sig**
**G**	15	11.54%	8	4.00%	0.01	kdPD	*
**L**	14	10.77%	9	4.50%	0.04	kdPD	*
**H**	3	2.31%	10	5.00%	0.26	kdSNP	
**I**	3	2.31%	11	5.50%	0.26	kdSNP	
**V**	6	4.62%	16	8.00%	0.27	kdSNP	
**Y**	5	3.85%	3	1.50%	0.27	kdPD	
**S**	4	3.08%	12	6.00%	0.3	kdSNP	
**W**	3	2.31%	1	0.50%	0.3	kdPD	
**D**	6	4.62%	15	7.50%	0.36	kdSNP	
**P**	4	3.08%	11	5.50%	0.42	kdSNP	
**A**	5	3.85%	12	6.00%	0.45	kdSNP	
**N**	4	3.08%	9	4.50%	0.58	kdSNP	
**M**	7	5.38%	8	4.00%	0.6	kdPD	
**R**	25	19.23%	35	17.50%	0.77	kdPD	
**T**	5	3.85%	10	5.00%	0.79	kdSNP	
**K**	7	5.38%	9	4.50%	0.8	kdPD	
**E**	8	6.15%	11	5.50%	0.81	kdPD	
**F**	2	1.54%	3	1.50%	1	kdPD	
**C**	3	2.31%	4	2.00%	1	kdPD	
**Q**	1	0.77%	3	1.50%	1	kdSNP	
	130	100.00%	200	100.00%			

kdPD: the kinase domain PDs (non redundant, nonsynonymous)
kdSNP: the kinase domain SNPs (non redundant, nonsynonymous)
n: the raw count of native residues of the particular amino acid in the corresponding dataset
%: the percentage of native residues of the particular amino acid in the corresponding dataset
p: the two-tailed Fisher exact test p-value
set: the set (kdPD/kdSNP) with the higher proportion of the particular amino acid as the native residue
sig: whether the p-value is significant (* for $p<0.05$, ** for $p<0.01$)

Table S2(b): Comparing kdPDs and kdSNPs with respect to mutant residue

The results of the Fisher exact tests comparing kdPDs and kdSNPs with respect to the mutant residue in the mutation/polymorphism pair. Results are ordered by ascending p-value.

	kdPD		kdSNP		statistics		
**Mutant**	**n**	**%**	**n**	**%**	**p**	**set**	**sig**
**P**	15	11.54%	2	1.00%	3.00E-005	kdPD	**
**L**	4	3.08%	15	7.50%	0.14	kdSNP	
**T**	5	3.85%	16	8.00%	0.17	kdSNP	
**W**	6	4.62%	4	2.00%	0.2	kdPD	
**M**	5	3.85%	15	7.50%	0.24	kdSNP	
**H**	6	4.62%	16	8.00%	0.27	kdSNP	
**F**	5	3.85%	4	2.00%	0.32	kdPD	
**G**	5	3.85%	4	2.00%	0.32	kdPD	
**E**	7	5.38%	6	3.00%	0.39	kdPD	
**V**	8	6.15%	18	9.00%	0.41	kdSNP	
**N**	3	2.31%	8	4.00%	0.54	kdSNP	
**I**	4	3.08%	9	4.50%	0.58	kdSNP	
**D**	7	5.38%	8	4.00%	0.6	kdPD	
**R**	10	7.69%	13	6.50%	0.67	kdPD	
**Q**	9	6.92%	17	8.50%	0.68	kdSNP	
**Y**	4	3.08%	5	2.50%	0.74	kdPD	
**A**	4	3.08%	5	2.50%	0.74	kdPD	
**K**	6	4.62%	8	4.00%	0.79	kdPD	
**S**	10	7.69%	17	8.50%	0.84	kdSNP	
**C**	7	5.38%	10	5.00%	1	kdPD	
	130	100.00%	200	100.00%			

kdPD: the kinase domain PDs (non redundant, nonsynonymous)
kdSNP: the kinase domain SNPs (non redundant, nonsynonymous)
n: the raw count of mutant residues of the particular amino acid in the corresponding dataset
%: the percentage of mutant residues of the particular amino acid in the corresponding dataset
p: the two-tailed Fisher exact test p-value
set: the set (kdPD/kdSNP) with the higher proportion of the particular amino acid as the mutant residue
sig: whether the p-value is significant (* for $p<0.05$, ** for $p<0.01$)

Table S2(c): Comparing kdPDs and kdSNPs with respect to native/mutant residue pair

The results of the Fisher exact tests comparing kdPDs and kdSNPs with respect to the mutation/polymorphism pairs observed. Results are ordered by ascending p-value.

cont...

	kdPD		kdSNP		statistics		
**Mutation**	**n**	**%**	**n**	**%**	**p**	**set**	**sig**
**L-P**	9	6.92%	0	0	0	kdPD	**
**K-E**	4	3.08%	0	0	0.02	kdPD	*
**R-P**	4	3.08%	0	0	0.02	kdPD	*
**G-R**	4	3.08%	1	0.50%	0.08	kdPD	
**K-R**	0	0	6	3.00%	0.08	kdSNP	
**D-N**	1	0.77%	7	3.50%	0.15	kdSNP	
**A-T**	1	0.77%	7	3.50%	0.15	kdSNP	
**L-R**	2	1.54%	0	0	0.15	kdPD	
**L-F**	2	1.54%	0	0	0.15	kdPD	
**A-D**	2	1.54%	0	0	0.15	kdPD	
**V-I**	0	0	4	2.00%	0.16	kdSNP	
**R-W**	5	3.85%	3	1.50%	0.27	kdPD	
**S-T**	0	0	3	1.50%	0.28	kdSNP	
**S-L**	0	0	3	1.50%	0.28	kdSNP	
**V-A**	0	0	3	1.50%	0.28	kdSNP	
**D-E**	0	0	3	1.50%	0.28	kdSNP	
**R-K**	0	0	3	1.50%	0.28	kdSNP	
**G-V**	3	2.31%	1	0.50%	0.3	kdPD	
**D-G**	3	2.31%	1	0.50%	0.3	kdPD	
**M-T**	3	2.31%	2	1.00%	0.39	kdPD	
**R-G**	1	0.77%	0	0	0.39	kdPD	
**I-K**	1	0.77%	0	0	0.39	kdPD	
**V-D**	1	0.77%	0	0	0.39	kdPD	
**V-F**	1	0.77%	0	0	0.39	kdPD	
**M-R**	1	0.77%	0	0	0.39	kdPD	
**N-T**	1	0.77%	0	0	0.39	kdPD	
**S-A**	1	0.77%	0	0	0.39	kdPD	

**R-I**	1	0.77%	0	0.00%	0.39	kdPD
**S-P**	1	0.77%	0	0.00%	0.39	kdPD
**K-N**	1	0.77%	0	0.00%	0.39	kdPD
**A-E**	1	0.77%	0	0.00%	0.39	kdPD
**M-K**	1	0.77%	0	0.00%	0.39	kdPD
**Y-D**	1	0.77%	0	0.00%	0.39	kdPD
**Y-F**	1	0.77%	0	0.00%	0.39	kdPD
**E-V**	1	0.77%	0	0.00%	0.39	kdPD
**Y-H**	1	0.77%	0	0.00%	0.39	kdPD
**C-R**	1	0.77%	0	0.00%	0.39	kdPD
**W-C**	1	0.77%	0	0.00%	0.39	kdPD
**F-S**	1	0.77%	0	0.00%	0.39	kdPD
**G-C**	1	0.77%	0	0.00%	0.39	kdPD
**D-Y**	1	0.77%	0	0.00%	0.39	kdPD
**W-S**	1	0.77%	0	0.00%	0.39	kdPD
**E-G**	1	0.77%	0	0.00%	0.39	kdPD
**Y-N**	1	0.77%	0	0.00%	0.39	kdPD
**G-A**	1	0.77%	0	0.00%	0.39	kdPD
**L-V**	1	0.77%	5	2.50%	0.41	kdSNP
**V-L**	1	0.77%	5	2.50%	0.41	kdSNP
**T-M**	2	1.54%	6	3.00%	0.49	kdSNP
**L-H**	0	0.00%	2	1.00%	0.52	kdSNP
**A-V**	0	0.00%	2	1.00%	0.52	kdSNP
**L-M**	0	0.00%	2	1.00%	0.52	kdSNP
**I-T**	0	0.00%	2	1.00%	0.52	kdSNP
**I-M**	0	0.00%	2	1.00%	0.52	kdSNP
**N-D**	0	0.00%	2	1.00%	0.52	kdSNP
**D-H**	0	0.00%	2	1.00%	0.52	kdSNP
**C-Y**	2	1.54%	1	0.50%	0.56	kdPD

cont...

**G-D**	2	1.54%	1	0.50%	0.56	kdPD
**R-H**	4	3.08%	10	5.00%	0.58	kdSNP
**G-S**	2	1.54%	2	1.00%	0.65	kdPD
**M-V**	1	0.77%	4	2.00%	0.65	kdSNP
**I-V**	1	0.77%	4	2.00%	0.65	kdSNP
**H-R**	1	0.77%	4	2.00%	0.65	kdSNP
**E-D**	1	0.77%	4	2.00%	0.65	kdSNP
**E-K**	3	2.31%	3	1.50%	0.68	kdPD
**N-S**	2	1.54%	5	2.50%	0.71	kdSNP
**R-Q**	6	4.62%	11	5.50%	0.8	kdSNP
**S-G**	0	0.00%	1	0.50%	1	kdSNP
**R-T**	0	0.00%	1	0.50%	1	kdSNP
**Y-S**	1	0.77%	2	1.00%	1	kdSNP
**V-E**	1	0.77%	1	0.50%	1	kdPD
**V-M**	2	1.54%	3	1.50%	1	kdPD
**W-R**	1	0.77%	1	0.50%	1	kdPD
**S-N**	0	0.00%	1	0.50%	1	kdSNP
**Y-C**	0	0.00%	1	0.50%	1	kdSNP
**T-I**	2	1.54%	3	1.50%	1	kdPD
**T-A**	1	0.77%	1	0.50%	1	kdPD
**S-C**	1	0.77%	2	1.00%	1	kdSNP
**S-F**	1	0.77%	2	1.00%	1	kdSNP
**A-P**	1	0.77%	1	0.50%	1	kdPD
**G-W**	1	0.77%	1	0.50%	1	kdPD
**H-D**	0	0.00%	1	0.50%	1	kdSNP
**F-Y**	0	0.00%	1	0.50%	1	kdSNP
**G-E**	1	0.77%	2	1.00%	1	kdSNP
**H-Q**	1	0.77%	1	0.50%	1	kdPD
**H-Y**	1	0.77%	2	1.00%	1	kdSNP

**H-L**	0	0.00%	1	0.50%	1	kdSNP
**H-P**	0	0.00%	1	0.50%	1	kdSNP
**C-G**	0	0.00%	1	0.50%	1	kdSNP
**C-S**	0	0.00%	1	0.50%	1	kdSNP
**A-S**	0	0.00%	1	0.50%	1	kdSNP
**C-F**	0	0.00%	1	0.50%	1	kdSNP
**E-Q**	1	0.77%	3	1.50%	1	kdSNP
**F-L**	1	0.77%	2	1.00%	1	kdSNP
**D-V**	1	0.77%	2	1.00%	1	kdSNP
**E-A**	1	0.77%	1	0.50%	1	kdPD
**I-F**	0	0.00%	1	0.50%	1	kdSNP
**P-S**	2	1.54%	4	2.00%	1	kdSNP
**P-T**	0	0.00%	1	0.50%	1	kdSNP
**P-L**	2	1.54%	4	2.00%	1	kdSNP
**P-Q**	0	0.00%	1	0.50%	1	kdSNP
**Q-R**	0	0.00%	1	0.50%	1	kdSNP
**R-C**	4	3.08%	7	3.50%	1	kdSNP
**Q-H**	1	0.77%	1	0.50%	1	kdPD
**Q-K**	0	0.00%	1	0.50%	1	kdSNP
**K-M**	1	0.77%	2	1.00%	1	kdSNP
**K-Q**	1	0.77%	1	0.50%	1	kdPD
**I-S**	1	0.77%	2	1.00%	1	kdSNP
**A-G**	0	0.00%	1	0.50%	1	kdSNP
**N-Y**	0	0.00%	1	0.50%	1	kdSNP
**P-H**	0	0.00%	1	0.50%	1	kdSNP
**M-I**	1	0.77%	2	1.00%	1	kdSNP
**N-K**	1	0.77%	1	0.50%	1	kdPD
	130	100.00%	200	100.00%		

cont...

kdPD: the kinase domain PDs (non redundant, nonsynonymous)
kdSNP: the kinase domain SNPs (non redundant, nonsynonymous)
n: the raw count of the particular mutation in the corresponding dataset
%: the percentage of the particular mutation in the corresponding dataset
p: the two-tailed Fisher exact test p-value
set: the set (kdPD/kdSNP) with the higher proportion of the particular mutation
sig: whether the p-value is significant (* for $p<0.05$, ** for $p<0.01$)

Table S3(a): Structural effect analysis comparing PDPK and SNPPK mutations

Fisher exact text results comparing the PDPK mutations with SNPPK mutations. Note that for both datasets, only the structure with the best SSAP identity score (see Methods) is considered when asking whether the mutation is explained by a given analysis. †: p≤0.05 / ‡ : p≤0.01.

	kdPDs (n=62)		kdSNPs (n=31)		statistics	
	n	%	n	%	p	†/‡
Functional	10	16.13	3	9.68	0.53	
Binding	8	12.9	3	9.68	0.75	
MMDB	2	3.23	0	0.00	0.55	
Folding	10	16.13	4	12.90	0.77	
Pro	3	4.84	0	0.00	0.55	
Gly	3	4.84	2	6.45	1.00	
Clash	6	9.68	2	6.45	0.71	
Cispro	0	0.00	0	0.00	1.00	
Instability	21	34.43	13	41.94	0.5	
Hbond	12	19.35	2	6.45	0.13	
Void	1	1.61	4	12.90	0.04	†
Corephilic	3	4.84	2	6.45	1.00	
Surfacephobic	6	9.68	3	9.68	1.00	
Buried charge	6	9.68	3	9.68	1.00	
SSgeom	0	0.00	0	0.00	1.00	
Interface(PQS)	17	27.42	2	6.45	0.03	†
SprotFT	12	19.35	5	16.13	0.78	
Sequence	6	9.68	1	3.23	0.42	
S/Explained	43	69.35	20	64.52	0.65	
Explained	45	72.58	20	64.52	0.48	

Table S3(b): Structural effect analysis comparing PDPK and PDnPK mutations

Fisher exact text results comparing the PDPK mutations with PDnPK mutations. Note that for PDnPKs the mutation is explained by a given analyses if at least one of the PDBs to which the mutations is mapped provides a positive explanation. For PDPKs, only the structure with the best SSAP identity score (see Methods) is considered. †: p≤0.05 / ‡ : p≤0.01).

	PDPK (n=62)		PDnPK (n=4652)		statistics	
	n	%	n	%	p	†/‡
Functional	10	16.13	1287	27.67	0.04	†
Binding	8	12.90	833	17.91	0.4	
MMDB	2	3.23	821	17.65	1.11-3	‡
Folding	10	16.13	906	19.48	0.63	
Pro	3	4.84	289	6.21	1	
Gly	3	4.84	270	5.80	1	
Clash	6	9.68	705	15.15	0.29	
Cispro	0	0.00	13	0.28	1	
Instability	21	34.43	2953	63.48	3.54-6	‡
Hbond	12	19.35	1237	26.59	0.25	
Void	1	1.61	1663	35.75	6.49-11	‡
Corephilic	3	4.84	270	5.80	1	
Surfacephobic	6	9.68	426	9.16	0.82	
Buried charge	6	9.68	591	12.70	0.57	
SSgeom	0	0.00	174	3.74	0.17	
Interface(PQS)	17	27.42	1716	36.89	0.14	
SprotFT	12	19.35	1322	28.42	0.12	
Sequence	6	9.68	928	19.95	0.05	
S/Explained	43	69.35	3867	83.13	9.49-3	‡
Explained	45	72.58	3954	85.00	1.15-2	†

Table S4: PD residues in the model explained by SAAPdb features

Model residue	Tree Determinants	Sequence Conservation	Structure Conservation	Buried	FireDB	Knight	Number of Features
**190**	X		X		X	X	4
**233**	X	X		X			3
**50**	X				X		2
**74**					X	X	2
**125**				X	X		2
**126**				X	X		2
**173**	X				X		2
**216**		X		X			2
**217**	X			X			2
**242**			X	X			2
**51**					X		1
**52**					X		1
**54**					X		1
**56**					X		1
**57**					X		1
**58**				X			1
**64**				X			1
**81**				X			1
**89**				X			1
**111**				X			1
**120**			X				1
**155**				X			1
**156**				X			1
**159**				X			1
**164**		X					1
**170**				X			1
**175**					X		1
**193**					X		1
**195**				X			1
**197**				X			1
**218**				X			1
**287**				X			1
**310**				X			1

Table S5: Comparing the present sequence analysis with those of others

Sequence Analysis results in comparison with previous works. Light yellow illustrates p-values<0.05 whereas Dark yellow illustrates more significant pvalues<0.01. X is used to denote non significant values.

Mutation type	PD_{PK}/SNP_{PK}	PD_{PK}/PD_{nPK}	Torkamani et al.	Hurst et al.
G-X	P=0.01 -> PDs	-	-	P=0.000 -> PDs
L-X	P=0.04 -> PDs	P=0.05 -> PDs	-	-
X-P	P=0.00 -> PDs	P=0.05 -> PDs	P=0.00 -> PDs	p=0.000 -> PDs
L-P	P=0.00 -> PDs	P=0.01 -> PDs	P=0.00 -> PDs	p=0.000 -> PDs
K-E	P=0.02 -> PDs	P=0.03 -> PDs	-	-
R-P	P=0.02 -> PDs	-	P=0.00 -> PDs	P=0.00 -> PDs

Supplementary Figures

Figure S1: Histograms of the distribution of distances between mutated residues and analyzed features for the pathogenic deviations (PDs) and neutral polymorphisms (SNPs).

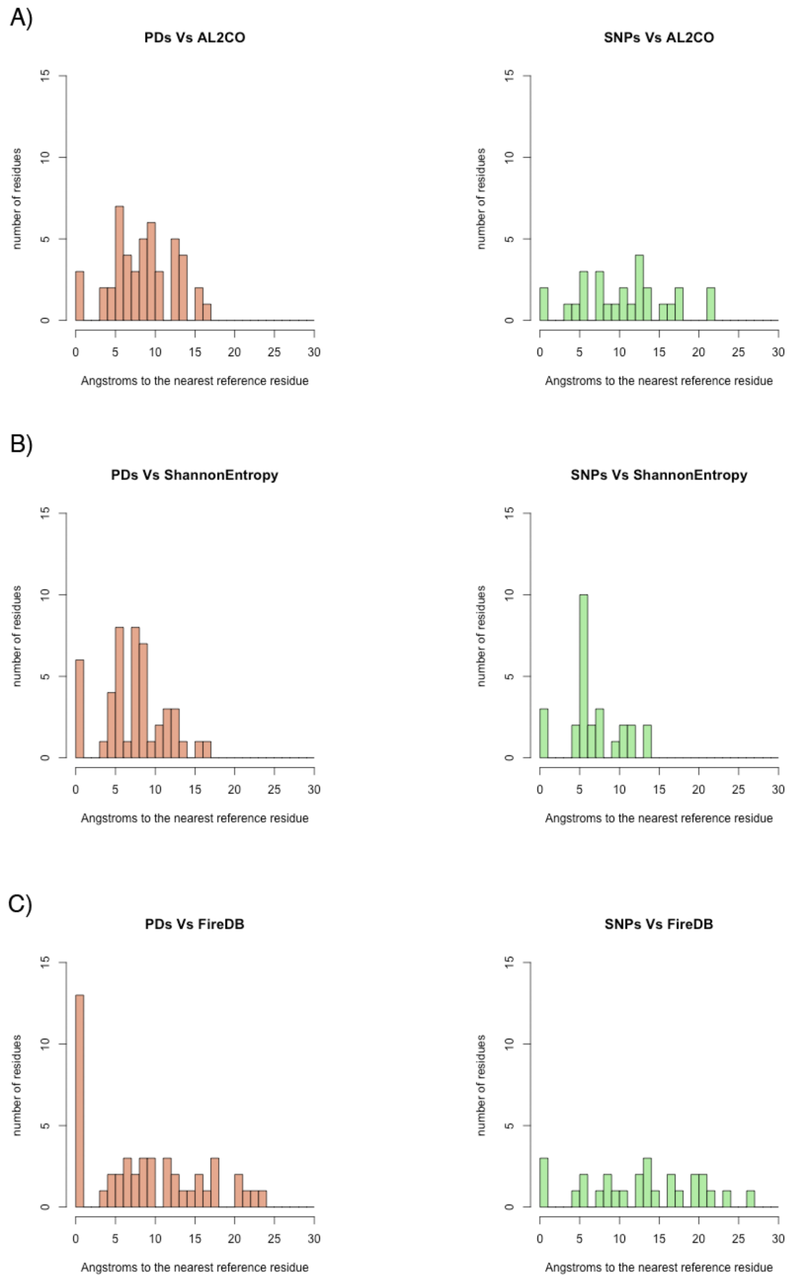

D)

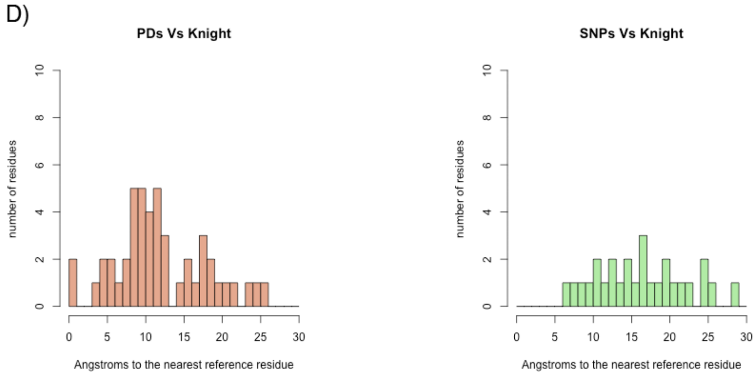
PDs Vs Knight
SNPs Vs Knight
number of residues
Angstroms to the nearest reference residue

E)

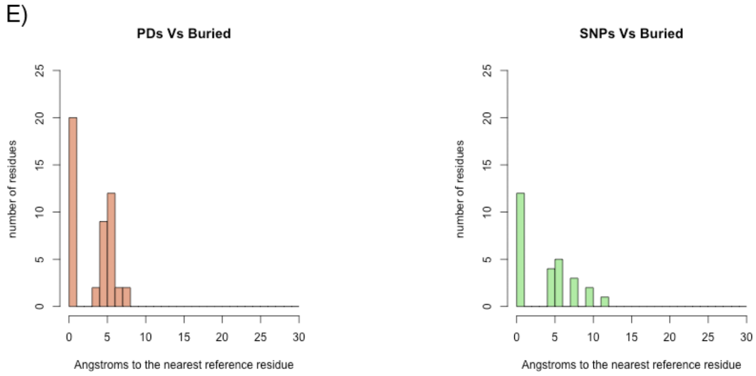
PDs Vs Buried
SNPs Vs Buried
number of residues
Angstroms to the nearest reference residue

F)

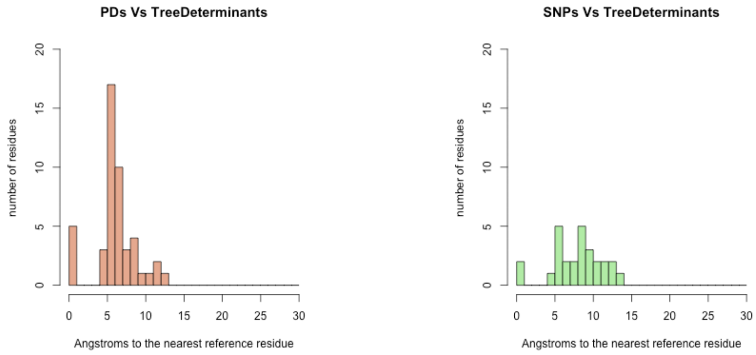
PDs Vs TreeDeterminants
SNPs Vs TreeDeterminants
number of residues
Angstroms to the nearest reference residue

G)

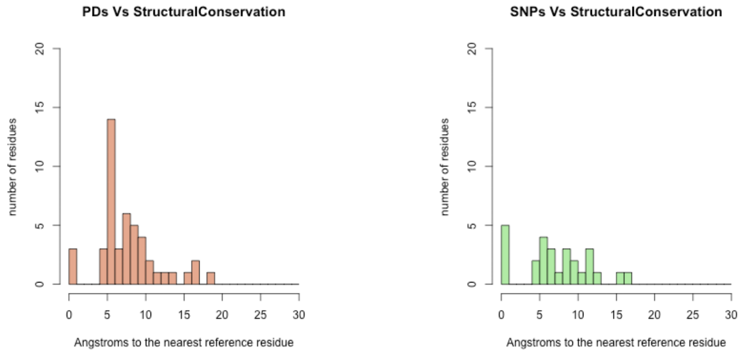
PDs Vs StructuralConservation
SNPs Vs StructuralConservation
number of residues
Angstroms to the nearest reference residue

Figure S2: Distribution of catalytic residues (yellow) as recorded in FireDB (Lopez et al, 2007), Pathogenic Deviations (PDs, light red) and neutral mutations (SNPs, light blue) within the model summarizing the structures of the Protein Kinase Domain. Residues that are both a catalytic residue and a PD are colored in dark red, whereas residues that are catalytic and neutrally mutated are represented in dark blue for comparison. Position 57 which was annotated both as PD and SNP is considered a pathogenic deviation in this figure. Spacefill is used to denote conserved residues in the catalytic core (Knight et al, 2007), i.e. K74, E96, D171, N176 and D190

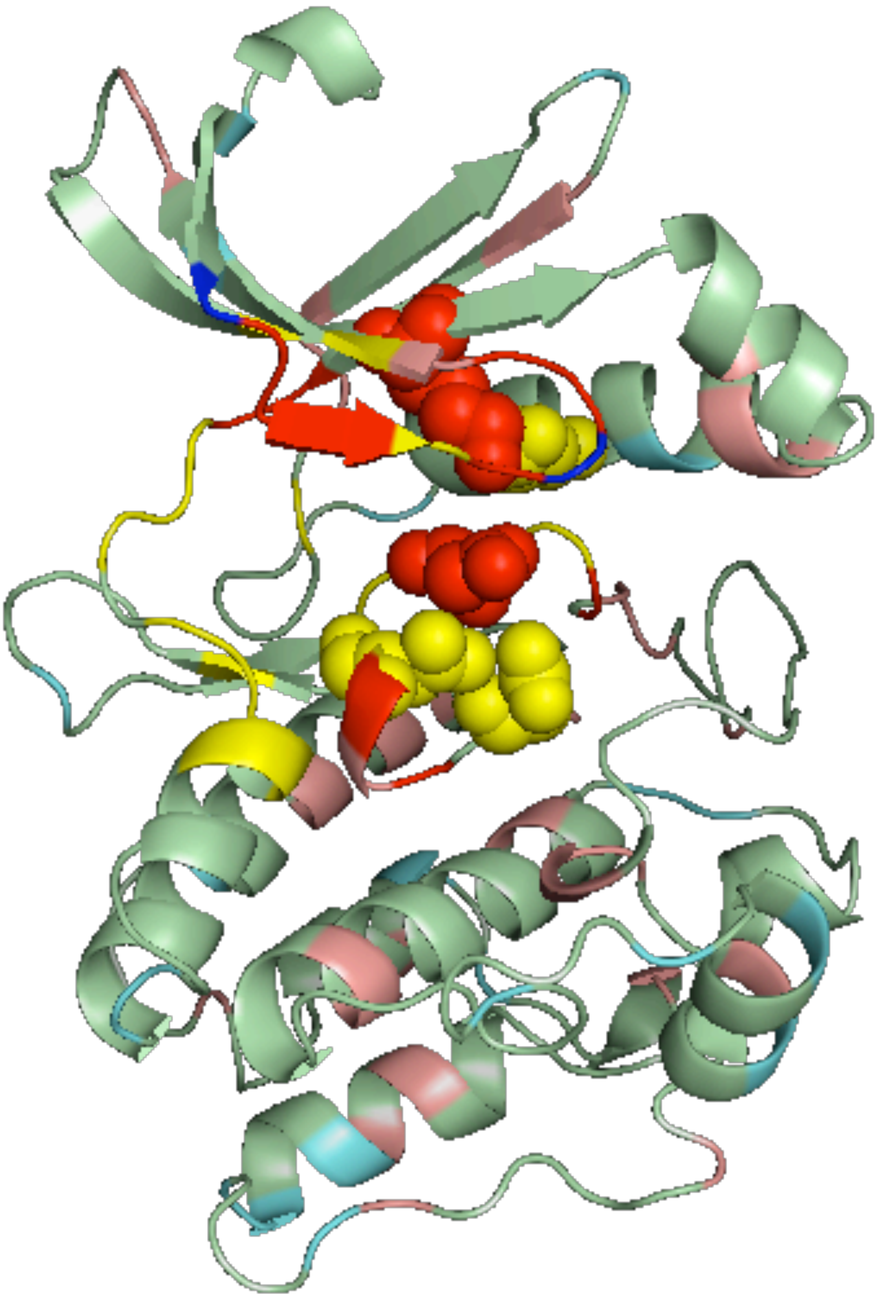

Figure S3: Distribution of Tree Determinant residues (yellow), Pathogenic Deviations (PDs, light red) and neutral mutations (SNPs, light blue) within the model summarizing the structures of the Protein Kinase Domain. Residues that are both a treedeterminant and a PD are colored in dark red, whereas residues that are a treedeterminant and neutrally mutated are represented in dark blue for comparison.

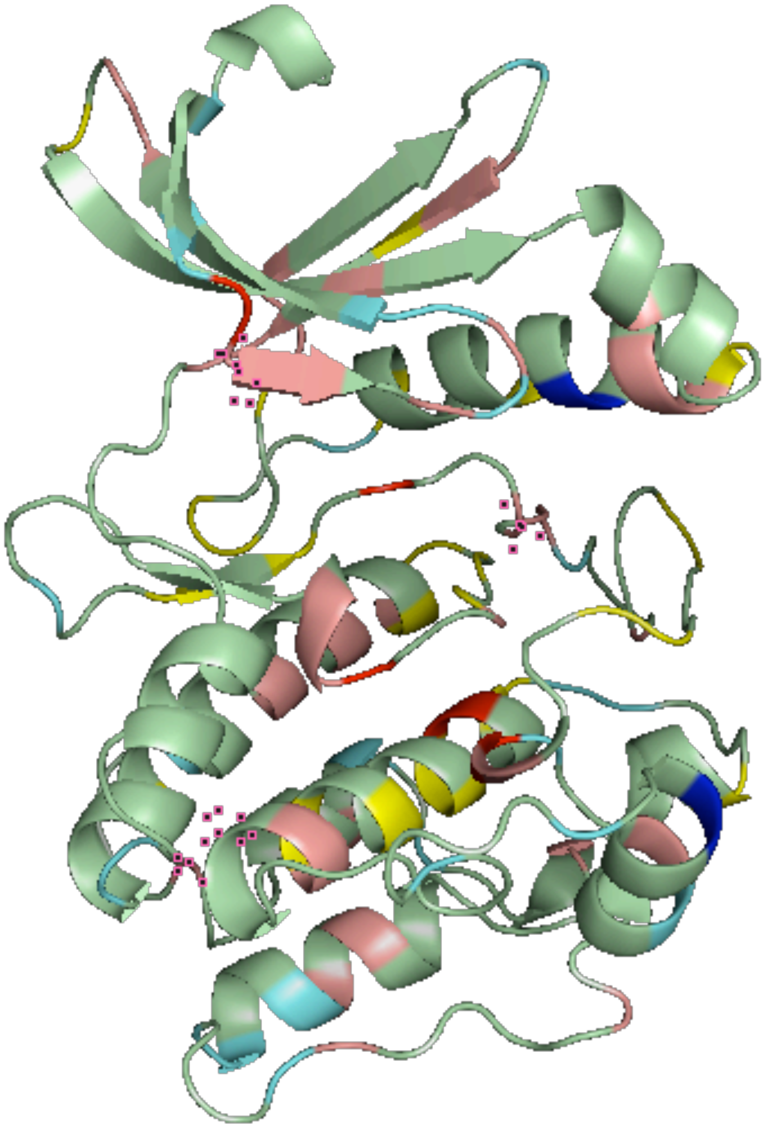

The most statistically informative tree-determinants are distributed in protein families as follows: four in the AGC sub-family, four in CK1, eight CMGC, and nine STE. The mapping of all those tree-determinant positions onto the representative structure is given in Figure S3. Visual inspection reveals many of the tree-determinants tend to locate near the ATP/substrate binding pocket of the protein and are potentially related with the differential binding properties. Other tree-determinant residues are located in regions for which the more plausible hypothesis is a role in the specific interaction with other partners and the participation in intramolecular signaling events (Dhillon et al., 2007).

Dhillon AS et al. (2007) MAP kinase signaling pathways in cancer. Oncogene 26, 3279-3290

Figure S4: Distribution of buried residues (yellow) calculated using Naccess (RSA<16%), Pathogenic Deviations (PDs, light red) and neutral mutations (SNPs, light blue) within the model summarizing the structures of the Protein Kinase Domain. Residues that are both buried and a PD are colored in dark red, whereas residues that buried and neutrally mutated are represented in dark blue for comparison. The position annotated as present in both PDs and neutral SNP datasets are colored in orange.

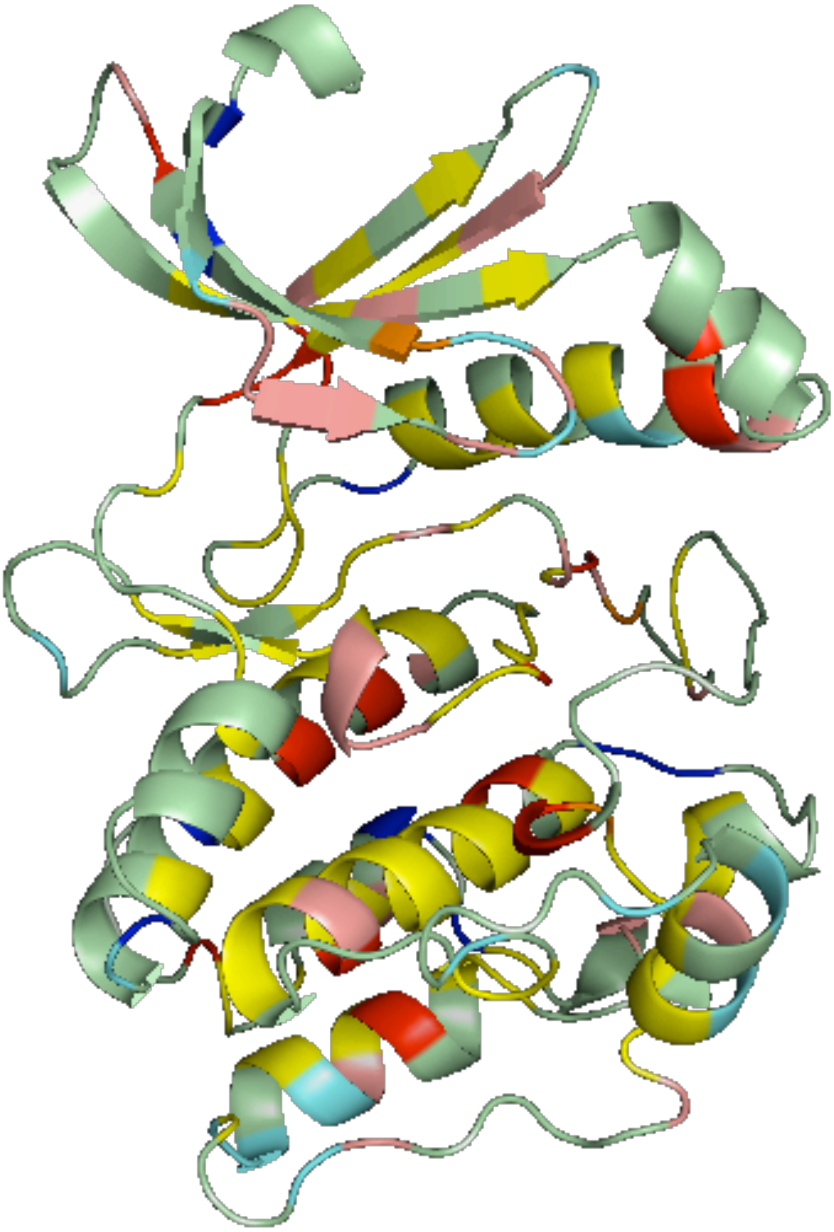

<u>Figure S5</u>: Distribution of conserved residues (yellow) evaluated in terms of variability using AL2CO (Pei et al, 2001), Pathogenic Deviations (PDs, light red) and neutral mutations (SNPs, light blue) within the model summarizing the structures of the Protein Kinase Domain. Residues that are both a conserved residue and a PD are colored in dark red, whereas residues that are conserved and neutrally mutated are represented in dark blue for comparison.

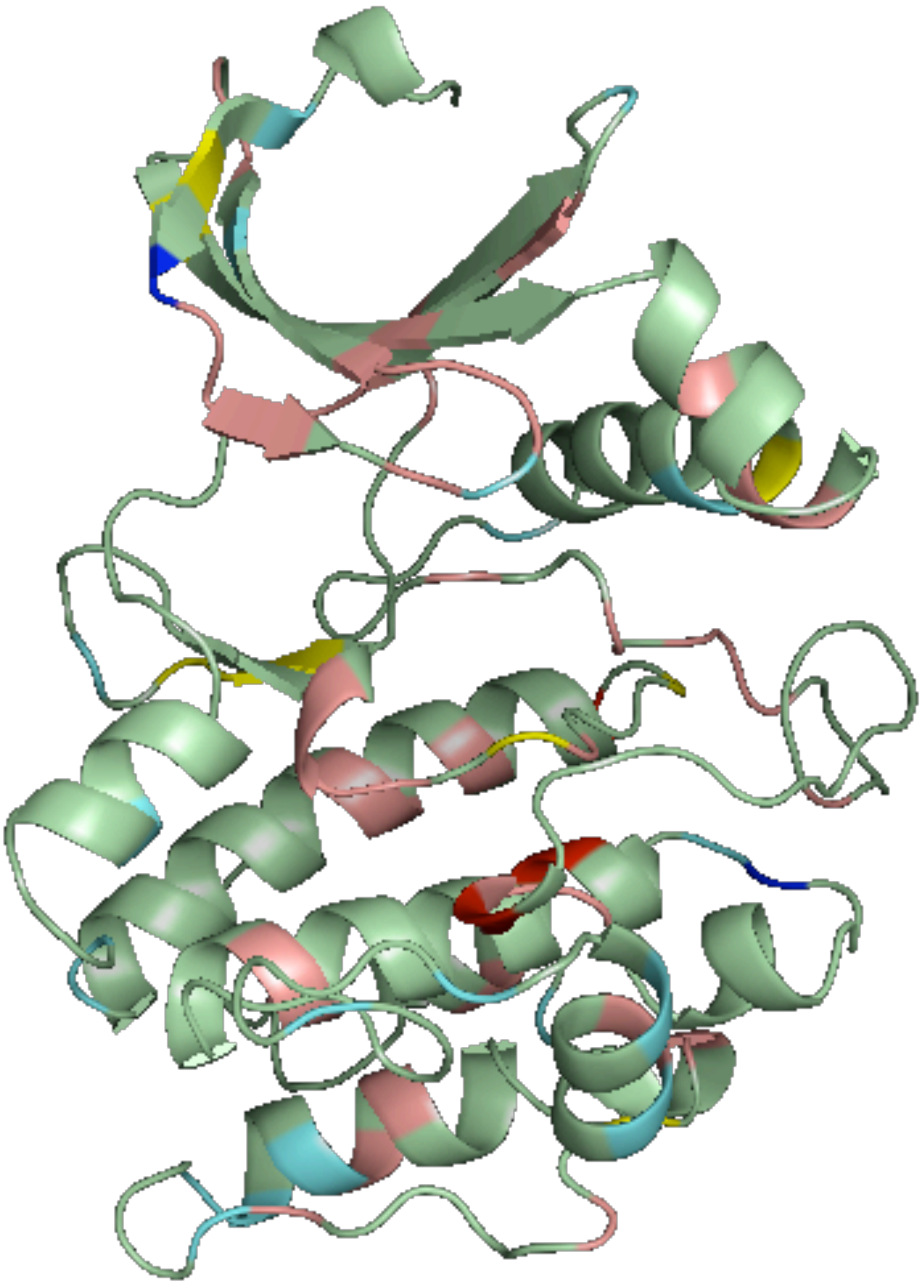

<u>Figure S6</u>: Distribution of structurally conserved residues (yellow) evaluated in terms of variability using CORA (Orengo, 1999), Pathogenic Deviations (PDs, light red) and neutral mutations (SNPs, light blue) within the model summarizing the structures of the Protein Kinase Domain. Residues that are both a conserved residue and a PD are colored in dark red, whereas residues that are conserved and neutrally mutated are represented in dark blue for comparison.

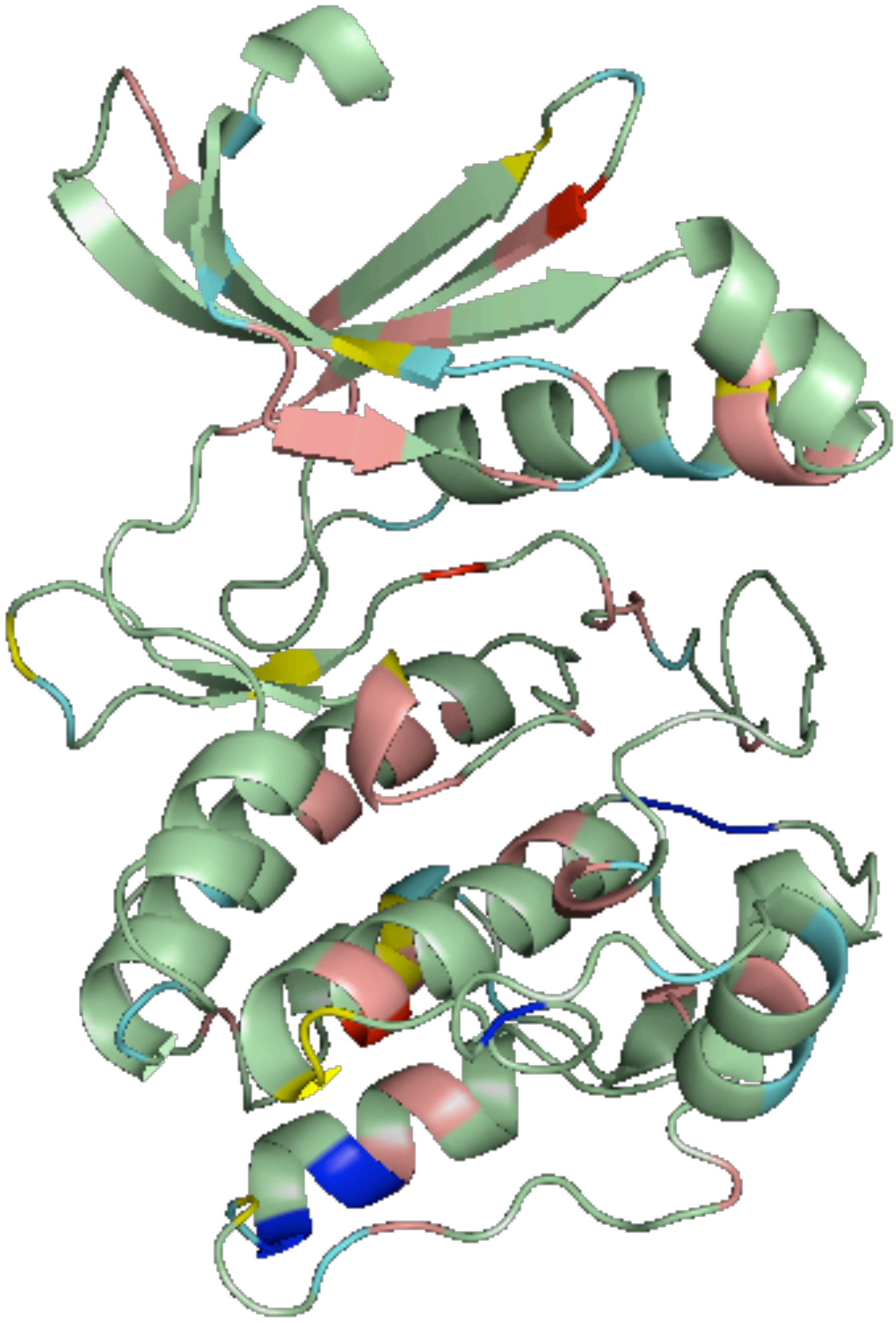

Supplement: Additional File 1 [file 1471-2105-12-S4-S1-S1.pdf]
